# Association between breakfast frequency and metabolic syndrome among young adults in South Korea

**DOI:** 10.1038/s41598-023-43957-3

**Published:** 2023-10-06

**Authors:** Hyo Myoung Kim, Hyun Jung Kang, Dong Hoon Lee, Su-Min Jeong, Hee-Kyung Joh

**Affiliations:** 1 Department of Family Medicine, Seoul National University Hospital, Seoul, Republic of Korea; 2 Department of Family Medicine, Samsung Medical Center, Sungkyunkwan University School of Medicine, Seoul, Republic of Korea; 3 Department of Sport Industry Studies, Yonsei University, Seoul, Republic of Korea; 4 Department of Nutrition, Harvard T.H. Chan School of Public Health, Boston, MA USA; 5 Department of Medicine, Seoul National University College of Medicine, 1, Gwanak-Ro, Gwanak-Gu, Seoul, 08826 Republic of Korea; 6 Department of Family Medicine, Seoul National University Health Service Center, Seoul, Republic of Korea

**Keywords:** Diseases, Medical research, Risk factors

## Abstract

Skipping breakfast is highly prevalent but it is not clear whether breakfast frequency is associated with metabolic syndrome in young adults. We aimed to assess the association between breakfast frequency and metabolic syndrome in Korean young adults. This cross-sectional study was based on health check-up data of university students aged 18–39 years between 2016 and 2018. Participants were stratified into three groups by breakfast frequency (non-skipper, skipper 1–3 days/week, skipper 4–7 days/week). Multivariable-adjusted logistic regression was used to calculate odds ratios (ORs) and 95% confidence intervals (CIs) of metabolic syndrome. Out of 12,302 participants, 56.8% skipped breakfast at least 4 days/week. Metabolic syndrome prevalence was higher in those skipping breakfast for 4 or more days/week compared to non-skipper. (3.1% vs 1.7%) In the age- and sex-adjusted model, individuals skipping breakfast for 4–7 days per week had a higher OR of metabolic syndrome (OR 1.73, 95% CI 1.21–2.49) compared to non-skipper. Although this association became insignificant (OR 1.49, 95% CI 0.99–2.23) after a fully adjusted multivariable model, trends of positive association between frequency of breakfast skipping and metabolic syndrome was significant (*P* for trend = 0.038). Frequent breakfast skipping was associated with higher odds of metabolic syndrome in young adults. Further longitudinal studies in the long term are needed to understand the association of meal patterns with metabolic syndrome.

## Introduction

Over the past, breakfast, the very first meal after the longest fasts has been regarded as the most important meal of the day in terms of body metabolism and circadian rhythm^1^. As modern lifestyle is moving away from regular meals, skipping breakfast is increasingly a common meal pattern in the current society^2^. According to Korea National Health and Nutrition Examination Survey data (2013–2018), 43.6% of participants reported eating breakfast less than 5 days/week^3^. In particular, more young adults (62.1%) did not have regular breakfast compared with middle-aged adults (33.9%).

Several previous studies reported that skipping breakfast has been associated with unfavorable health outcomes such as obesity, type 2 diabetes mellitus, and hypertension^4–6^. For example, breakfast skipping was associated with higher glycemic responses after lunch compared to when breakfast was consumed in healthy young men^7^. A recent meta-analysis reported that skipping breakfast was associated with an increased risk of hypertension (hazard ratio = 1.20, 95% CI 1.08–1.33)^8^. This association was consistently observed across countries, study design, and definition of breakfast skippers. An Australian longitudinal study reported long-term detrimental effects of skipping breakfast during childhood (9–15 years old) on cardio-metabolic health later in adulthood (26–36 years old)^9^. A larger waist circumference and higher low-density lipoprotein cholesterol (LDL-C) levels in adulthood was noted among those who skipped breakfast in childhood. In addition, skipping breakfast was associated with higher calorie ingestion later in the evening^10^ and less optimal nutrient intake^11^, which may impact metabolic parameters.

Based on Korea National Health and Nutrition Examination Survey data (2016–2018), the prevalence of metabolic syndrome was 23% among Korean adults aged ≥ 19 years. The prevalence of metabolic syndrome in men over 30 years of age has sharply increased^12^. A number of studies have demonstrated significant positive associations between skipping breakfast and metabolic syndrome^3,6,13,14^, while other studies found opposite association between skipping breakfast and metabolic syndrome^15^. However, some studies did not consider diet quality^3,14,15^ and other meal patterns (binge eating or irregularity of meal eating)^6,13^. Most of previous studies encompassed broad age groups without focusing on young adults. Early adulthood is a critical period of life transition, including leaving the parental home for further education or marriage, which may disrupt an individual’s pre-existing habits and make changes in diet and dietary behaviors^16^. Moreover, adopting and maintaining healthy lifestyle such as healthy diet in young adulthood could be linked to a lower risk of subsequent cardiovascular diseases in middle age^17^.

Therefore, investigating the association between skipping breakfast and prevalence of metabolic syndrome and its components in young adults can add evidence on the importance of healthy meal patterns in young adulthood. In this study, we primarily aimed to evaluate the association between skipping breakfast and metabolic syndrome in young adults. Additionally, we evaluated the association between general meal patterns and metabolic syndrome.

## Materials and methods

### Study population

We used health check-up data of 15,381 Seoul National University students, including undergraduate, graduate, and doctoral students, from 2016 through 2018. All students were eligible to voluntarily have health check-up for free once a year, and the health screening included a self-reported questionnaire (health behavior), anthropometric measurements (height, weight, and waist circumference), and laboratory tests^18^. We excluded participants who (1) were under 18 years or over 40 years of age (n = 137), (2) declined to participate in the study (n = 638), (3) were taking medication for hypertension, diabetes mellitus, or dyslipidemia (n = 45), (4) were foreigners (n = 1094), (5) were pregnant (n = 19), or (6) had incomplete information (n = 1146). Ultimately, a total of 12,302 participants were included in this study. All individuals provided informed consent to participate in the study, which was approved by the Institutional Review Board of Seoul National University (IRB number, C-1304–062-481). Research involving human research participants must have been performed in accordance with the Declaration of Helsinki.

### Anthropometric and laboratory measurements

Weight and height were measured with participants in light clothing on the day of health check-up. Body mass index (BMI) was calculated as weight (kg) divided by the square of the height (m^2^). Waist circumference was measured at the midpoint between the last rib and the top of the iliac crest. Blood pressure (BP) measurements were taken with the participant in a sitting position using an automatic BP measurement system after a rest period of at least 5 min. Blood samples were taken after at least 12 h of fasting.

### Definition of metabolic syndrome

The definition of metabolic syndrome was taken from the modified National Cholesterol Education Program (NCEP) Adult Treatment Panel III guidelines^19^. Subjects who met three or more of the following criteria were diagnosed with metabolic syndrome: (1) central obesity (waist circumference ≥ 90 cm for men or ≥ 85 cm for women); (2) hypertriglyceridemia with fasting plasma triglyceride levels ≥ 150 mg/dL; (3) decreased levels of high density lipoprotein cholesterol (HDL-C) with HDL-C levels < 40 mg/dL for men and < 50 mg/dL for women; (4) hypertension with systolic or diastolic BP ≥ 130/85 mmHg; and (5) hyperglycemia with fasting plasma glucose ≥ 100 mg/dL.

### Assessment of breakfast eating, meal patterns and dietary intake

Using a self-administered questionnaire, participants were asked how often, on average, they consumed breakfast during the past year: “How often do you eat breakfast?” According to the answers, the participants were then categorized into three groups: having breakfast for 7 days per week (non-skipper), 4–6 days per week (skipper for 1–3 days), and 0–3 days per week (skipper for 4–7 days).

Using the question “How often do you overeat or binge?”, we divided participants into three groups based on frequency of binge eating per week: less than once per week (e.g., 2–3 times per month), 1–2 times per week, and ≥ 3 times per week.

To assess meal frequency per day, we categorized the participants into two groups, using the question “How many meals do you usually eat a day?”: regular (3 meals per day) and irregular (< 3 meals per day).

Overall meal pattern was defined by combination of three variables: breakfast frequency per week (> 3 days per week or ≤ 3 days per week), binge eating per week (< 3 times per week or ≥ 3 times per week), and meal frequency per day (3 meals per day or < 3 meals per day on average). An “unhealthy” meal pattern was defined if an individual had all the bad eating patterns; “fair” was defined if an individual had 1 or 2 bad eating patterns; and “healthy” was defined if a participant had no bad eating patterns. For example, someone had an unhealthy meal pattern if they had 0–3 days with breakfast per week, ≥ 3 times of binge eating per week, and < 3 meals per day.

We also evaluated dietary intake of various foods by asking consumption frequency of fruits, vegetables, milk and dairy products, high-fat meat, processed meat, and sugared beverages (< once a week, 1–4 times a week, ≥ 5 times a week).

### Other variables

Alcohol consumption was classified into non-drinker, moderate drinker, and heavy drinker based on self-questionnaires. Moderate drinking was defined as consuming 14 or less standard drinks per week in men and 7 or less standard drinks per week in women, and heavy drinking was defined as more than these amounts (1 standard drink = 12 g of alcohol)^20^. Smoking status was categorized into never, ex-smoker, and current smoker based on self-questionnaires. Physical activity levels were evaluated using the International Physical Activity Questionnaire and total physical activity levels (metabolic equivalent [MET]-min/week) were categorized into low (< 600 MET-min/week), moderate (600–2999 MET-min/week), and high (≥ 3000 MET-min/week) physical activity^21^.

### Statistical analysis

Data were expressed as the mean with standard deviations (SD) for continuous variables or as number with percentages for categorical variables. χ^2^ and the analysis of variance for categorical and continuous variables were used to compare general characteristics of the study population by breakfast consumption status. Logistic regression analyses were performed to estimate odds ratios (ORs) and 95% confidence intervals (CIs) of metabolic syndrome, with adjustment for various lifestyle and dietary factors. Model 1 was adjusted for age and sex, and model 2 was adjusted for alcohol consumption, smoking status, physical activity, and BMI in addition to the covariates of model 1. Model 3 was adjusted for dietary intake of fruits, vegetables, milk and dairy products, high-fat meat, processed meat, and sugared beverages in addition to the covariates of model 2. All statistical analyses were performed using Stata ver. 16.0 for Windows (Stata Corp., College Station, TX, USA). P < 0.05 was considered statistically significant.

## Results

### General characteristics of study population

Table 1 shows general characteristic differences across the three groups by breakfast frequency. The mean age was 24.0 ± 3.9 years. Breakfast skippers for 4–7 days/week tended to be older (24.1 years), male (55.9%), heavy drinkers (18.3%), current smokers (9.4%), and physically inactive (19.5%). The prevalence of obesity was higher in breakfast skippers for 4–7 days/week than non-skippers (16.2% vs. 13.7%). The participants who had binge eating for 3 times or more per week (7.8%) and irregular meal (86.5%) were more prevalent among those who skipped breakfast for 4–7 days/week than other groups. Non-skippers more frequently consumed brown rice, multigrain rice, beans, fruits, vegetables, milk and dairy products, egg, and coffee, and less frequently consumed bread/toast, pizza, hamburger or sandwich, rice cake/Tteokbokki, instant noodles or cup ramen, and sugared beverages (Supplementary Table 1).

**Table 1:** General characteristics of study participants according to breakfast consumption frequency.

	Total	Breakfast frequency, per week			p-value
		7 days (non-skipper)	4–6 days (skipper for 1–3 days)	0–3 days (skipper for 4–7 days)	
	n = 12,302	n = 2152 (17.5%)	n = 3169 (25.8%)	n = 6981 (56.8%)	
Age,	24.0 ± 3.9	23.8 ± 4.1	23.9 ± 3.9	24.1 ± 3.8	< 0.001
Sex					< 0.001
Male	6579 (53.4)	1063 (49.4)	1671 (52.7)	3845 (55.9)	
Female	5723 (46.5)	1089 (50.6)	1498 (47.3)	3136 (44.9)	
Alcohol consumption					< 0.001
None	887 (7.2)	264 (12.3)	209 (6.6)	414 (5.9)	
Moderate	9483 (77.1)	1653 (76.8)	2543 (80.3)	5287 (75.7)	
Heavy	1932 (15.7)	235 (10.9)	417 (13.2)	1280 (18.3)	
Smoking status					< 0.001
Never	10,676 (86.8)	1982 (92.1)	2810 (88.7)	5884 (84.3)	
Ex-smoker	723 (5.9)	101 (4.7)	180 (5.7)	442 (6.3)	
Current smoker	903 (7.3)	69 (3.2)	179 (5.7)	655 (9.4)	
Physical activity					< 0.001
Low	2019 (16.4)	227 (10.6)	434 (13.7)	1358 (19.5)	
Moderate	8370 (68.0)	1481 (68.8)	2177 (68.7)	4712 (67.5)	
High	1913 (15.6)	444 (20.6)	558 (17.6)	911 (13.1)	
Body mass index, kg/m^2^	22.0 ± 3.1	21.9 ± 2.9	22.0 ± 3.0	22.1 ± 3.2	< 0.001
Underweight (< 18.5)	1201 (9.8)	215 (10.0)	307 (9.7)	679 (9.7)	0.004
Normal (18.5–23)	7084 (57.6)	1287 (59.8)	1863 (58.8)	3934 (56.4)	
Overweight (23–25)	2161 (17.6)	355 (16.5)	568 (17.9)	1239 (17.8)	
Obese (≥ 25)	1855 (15.1)	295 (13.7)	431 (13.6)	1129 (16.2)	
Binge eating frequency, per week					< 0.001
< 1	8181 (66.5)	1573 (73.1)	2179 (68.8)	4429 (63.4)	
1–2	3259 (26.5)	465 (21.61)	787 (24.8)	2007 (28.8)	
≥ 3	862 (7.0)	114 (5.3)	203 (6.4)	545 (7.8)	
Meal frequency, per day					< 0.001
Regular (3 meals per day)	5334 (43.4)	1924 (89.4)	2465 (77.8)	945 (13.5)	
Irregular (< 3 meals per day)	6968 (56.6)	228 (10.6)	704 (22.2)	6036 (86.5)	

Values are presented as mean ± standard deviation or number (%).

*SD* standard deviation.

### Prevalence of abnormal metabolic parameter by breakfast consumption status

The mean value or prevalence of abnormal metabolic parameters are presented in Table 2**.** Each component of metabolic syndrome as well as hyperuricemia were more prevalent among participants skipping breakfast more frequently. Participants who frequently skipped breakfast for 4–7 days/week were more likely to have metabolic syndrome compared with non-skipper (3.1% vs. 1.7%; p < 0.001).

**Table 2:** Metabolic parameters of study participants according to breakfast consumption frequency.

	Total	Breakfast skipping, per week			p-value
		Non-skipper	Skipper for 1–3 days	Skipper for 4–7 days	
	n = 12,302	n = 2152 (17.5%)	n = 3169 (25.8%)	n = 6981 (56.8%)	
Waist circumference, cm, mean ± SD	76.0 ± 9.0	75.4 ± 8.6	75.6 ± 8.6	76.3 ± 9.2	< 0.001
Normal (Male < 90, Female < 85)	11,310 (91.9)	2003 (93.1)	2959 (93.4)	6348 (90.9)	< 0.001
Abdominal obesity (Male ≥ 90, Female ≥ 85)	992 (8.1)	149 (6.9)	210 (6.6)	633 (9.1)	
Blood pressure, mmHg					
Systolic BP, mean ± SD	111.9 ± 12.5	110.1 ± 12.6	111.4 ± 12.4	112.7 ± 12.4	< 0.001
Diastolic BP, mean ± SD	66.8 ± 8.7	65.3 ± 8.7	66.4 ± 8.6	67.4 ± 8.7	< 0.001
Systolic BP ≥ 130 or Diastolic BP ≥ 85, N (%)	1104 (9.0)	153 (7.1)	265 (8.4)	686 (9.8)	< 0.001
Laboratory test, mg/dL					
Fasting glucose, mean ± SD	90.7 ± 7.7	89.7 ± 6.8	90.5 ± 7.6	91.1 ± 8.0	< 0.001
≥ 100, N(%)	1151 (9.4)	157 (7.3)	280 (8.8)	714 (10.2)	< 0.001
Total cholesterol, mean ± SD	176.8 ± 29.6	174.7 ± 30.8	175.2 ± 29.1	178.2 ± 29.5	< 0.001
≥ 200, N(%)	2436 (19.8)	392 (18.2)	587 (18.5)	1457 (20.9)	0.003
Triglycerides, mean ± SD	86.5 ± 48.7	81.4 ± 47.5	83.7 ± 44.7	89.3 ± 50.6	< 0.001
≥ 150, N(%)	896 (7.3)	123 (5.7)	195 (6.2)	578 (8.3)	< 0.001
HDL-C, mean ± SD	65.8 ± 15.4	66.7 ± 15.5	65.8 ± 15.4	65.4 ± 15.4	0.004
Male < 40, Female < 50, N (%)	546 (4.4)	83 (3.9)	133 (4.2)	330 (4.7)	0.172
LDL-C, mean ± SD	93.8 ± 26.7	91.9 ± 27.1	92.8 ± 26.0	95.0 ± 26.9	< 0.001
≥ 100, N(%)	4,550 (37.0)	706 (32.8)	1137 (35.9)	2707 (38.8)	< 0.001
Uric acid, mean ± SD	5.3 ± 1.4	5.2 ± 1.3	5.3 ± 1.3	5.4 ± 1.4	< 0.001
Male ≥ 7, Female ≥ 6, N (%)	1709 (13.9)	253 (11.8)	432 (13.6)	1024 (14.7)	0.003
Number of MS components, N (%)					
0	9067 (73.7)	1664 (77.3)	2404 (75.9)	4999 (71.6)	< 0.001
1	2190 (17.8)	352 (16.4)	535 (16.9)	1303 (18.7)	< 0.001
2	726 (5.9)	100 (4.7)	165 (5.2)	461 (6.6)	< 0.001
≥ 3 (MS)	319 (2.6)	36 (1.7)	65 (2.1)	218 (3.1)	< 0.001

*BP* blood pressure, *HDL-C* high-density lipoprotein cholesterol, *LDL-C* low-density lipoprotein cholesterol, *MS* metabolic syndrome, *SD* standard deviation.

### Association between breakfast consumption status and metabolic syndrome

The associations of breakfast consumption with metabolic syndrome and its components are shown in Table 3. In model 2, frequent breakfast skipping for 4–7 days/week was associated with a higher prevalence of metabolic syndrome (OR 1.61, 95% CI 1.09–2.36) compared to non-breakfast skipping. After additional adjustment for dietary intake in model 3, the association between frequent breakfast skipping for 4–7 days/week and metabolic syndrome was no longer significant (OR 1.49, 95% CI 0.99–2.33). However, there was a significant positive trend of the association between frequency of skipping breakfast and metabolic syndrome (*P* trend = 0.038). Among components of metabolic syndrome, breakfast skipper for 4–7 days/week had higher OR of high BP than non-skipper (OR: 1.34, 95% CI 1.09–1.65). The association between frequency of skipping breakfast and metabolic syndrome was consistent with main results when we applied to International Diabetes Federation- and World Health Organization-defined metabolic syndrome (Supplementary Table 2).

**Table 3:** Multivariable-adjusted odds ratios and 95% confidence intervals for metabolic syndrome and its components according to breakfast consumption frequency.

	Breakfast frequency, per week			*p*_trend_
	Non-skipper	Skipper for 1–3 days	Skipper for 4–7 days	
Waist circumference, Male ≥ 90, Female ≥ 85 (cm)				
Model 1	1 (Ref)	0.91 (0.73–1.14)	1.24 (1.02–1.49)	0.002
Model 2	1 (Ref)	0.92 (0.70–1.22)	1.13 (0.88–1.44)	0.147
Model 3	1 (Ref)	0.91 (0.68–1.20)	1.08 (0.84–1.40)	0.312
Systolic BP ≥ 130 or Diastolic BP ≥ 85 (mmHg)				
Model 1	1 (Ref)	1.13 (0.92–1.40)	1.30 (1.08–1.57)	0.003
Model 2	1 (Ref)	1.17 (0.94–1.46)	1.30 (1.07–1.57)	0.008
Model 3	1 (Ref)	1.19 (0.96–1.49)	1.34 (1.09–1.65)	0.005
Fasting glucose ≥ 100 (mg/dL)				
Model 1	1 (Ref)	1.19 (0.97–1.47)	1.35 (1.13–1.63)	0.001
Model 2	1 (Ref)	1.18 (0.96–1.45)	1.28 (1.06–1.54)	0.010
Model 3	1 (Ref)	1.13 (0.91–1.40)	1.15 (0.95–1.41)	0.199
Triglycerides ≥ 150 (mg/dL)				
Model 1	1 (Ref)	1.04 (0.82–1.32)	1.38 (1.13–1.70)	< 0.001
Model 2	1 (Ref)	1.01 (0.79–1.29)	1.22 (0.99–1.51)	0.021
Model 3	1 (Ref)	0.98 (0.76–1.25)	1.09 (0.87–1.37)	0.286
HDL-C < 40, male / < 50, female (mg/dL)				
Model 1	1 (Ref)	1.09 (0.83–1.45)	1.23 (0.96–1.58)	0.069
Model 2	1 (Ref)	1.08 (0.82–1.45)	1.12 (0.87–1.45)	0.400
Model 3	1 (Ref)	1.08 (0.81–1.45)	1.15 (0.88–1.50)	0.312
1–2 of metabolic syndrome components				
Model 1	1 (Ref)	1.03 (0.90–1.19)	1.22 (1.08–1.38)	< 0.001
Model 2	1 (Ref)	1.01 (0.88–1.16)	1.15 (1.02–1.30)	0.007
Model 3	1 (Ref)	0.99 (0.86–1.14)	1.09 (0.95–1.24)	0.312
Metabolic syndrome				
Model 1	1 (Ref)	1.18 (0.78–1.80)	1.73 (1.21–2.49)	< 0.001
Model 2	1 (Ref)	1.26 (0.81–1.95)	1.61 (1.09–2.36)	0.008
Model 3	1 (Ref)	1.23 (0.79–1.91)	1.49 (0.99–2.23)	0.038

Values are presented as number (%) or mean (95% confidence interval). Logistic regression analysis was performed to the estimate odds ratios (ORs) and 95% confidence intervals (CIs).

Model 1 adjusted for age, and sex.

Model 2 adjusted for age, sex, alcohol, smoking, physical activity, and BMI.

Model 3 adjusted for age, sex, alcohol, smoking, physical activity, BMI, and dietary intake of fruits, vegetables, milk and dairy products, high-fat meat, processed meat, and sugared beverages.

*BP* blood pressure, *HDL-C* high density lipoprotein-cholesterol, *LDL-C* low density lipoprotein-cholesterol, *OR* odds ratio, *CI* confidence interval.

In stratification analyses by sex and physical activity, there was no significant interaction with skipping breakfast. (Supplementary Tables 3 and 4).

### Association between overall meal patterns and metabolic syndrome

Table 4 shows the association between overall meal patterns and metabolic syndrome. The ORs of metabolic syndrome increased as the group had unhealthier meal patterns compared to the group with a healthy meal pattern (OR 1.80, 95% CI 1.02–3.19 for unhealthy; OR 1.55, 95% CI 0.90–2.66 for fair meal pattern, P for trend = 0.055).

**Table 4:** Multivariable-adjusted odds ratios and 95% confidence intervals for metabolic syndrome according to the meal pattern.

	Meal pattern					*p*_trend_
	Healthy	Fair		Unhealthy		
	n = 1421	n = 8454	p-value	n = 2427	p-value	
N (%)	17 (1.2)	188 (2.2)		114 (4.7)		
Model 1	1 (Ref)	1.89 (1.14–3.13)	0.013	4.03 (2.40–6.78)	< 0.001	< 0.001
Model 2	1 (Ref)	1.64 (0.96–2.78)	0.068	1.99 (1.15–3.46)	0.014	0.013
Model 3	1 (Ref)	1.55 (0.90–2.66)	0.11	1.80 (1.02–3.19)	0.044	0.055

Overall meal pattern was defined by combination of three variables: breakfast frequency per week (> 3 days per week or ≤ 3 days per week), binge eating per week (< 3 times per week or ≥ 3 times per week), and meal frequency per day (3 meals per day or < 3 meals per day on average). An “unhealthy” meal pattern was defined if an individual had all the bad eating patterns; “fair” was defined if an individual had 1 or 2 bad eating patterns; and “healthy” was defined if a participant had no bad eating patterns.

Model 1 adjusted for age, sex.

Model 2 adjusted for age, sex, alcohol, smoking, physical activity, BMI.

Model 3 adjusted for age, sex, alcohol, smoking, physical activity, BMI, and dietary intake of fruits, vegetables, milk and dairy products, high-fat meat, processed meat, and sugared beverages.

In the case of binge eating, the OR of metabolic syndrome increased as the frequency of binge eating per week increased, but there was no statistical significance when fully adjusted (OR 1.14, 95% CI 0.87–1.49 for 1–2 times of binge eating/week; OR 1.18, 95% CI 0.80–1.74 for ≥ 3 times of binge eating/week) (Supplementary Table 5). Compared to the group who had three meals per day, the OR of metabolic syndrome increased in the case of irregular meals, with marginal statistical significance when fully adjusted (OR 1.26, 95% CI 0.97–1.64, p = 0.084 for irregular meals per day) (Supplementary Table 6).

## Discussion

In this cross-sectional study among Korean young adults, we found a significant positive trend between breakfast skipping frequency and metabolic syndrome (P for trend = 0.038) and its components, especially high BP. Overall unhealthy meal pattern (defined by combination of breakfast skipping, binge eating, and irregular meal) was associated with higher odds of metabolic syndrome compared with healthy meal pattern.

Prior studies found similar results that skipping breakfast was associated with poorer cardio-metabolic health^22^. A recent meta-analysis based on observational studies reported that individuals having breakfast 3 times/week or more had lower risk of type 2 diabetes mellitus, hypertension, obesity, low LDL-C, and metabolic syndrome^22^. In a longitudinal study of Health Professionals Follow-up study (26,902 men with 16 years of follow-up) reported that those who skipped breakfast had a 27% higher risk of coronary heart disease compared with non-skippers^23^.

Several biological mechanisms underlying the positive association between skipping breakfast and metabolic syndrome could be suggested. First, skipping breakfast can impair insulin sensitivity. In a randomized crossover trial among healthy women, skipping breakfast impaired postprandial insulin sensitivity^24^. After skipping breakfast, the curve of serum insulin responses rose, whereas it fell significantly after eating breakfast. The ‘second-meal phenomenon’ could be related to impaired insulin sensitivity after breakfast skipping, which means prior meal can influence glycemic responses to a subsequent meal^7^. Plasma glucose after lunch rose less when breakfast was taken via suppressed free fatty acids and muscle glycogen syntheses^25^. Second, elevated insulin levels related to breakfast skipping can cause weight gain. A prospective cohort study in the US reported that eating breakfast was associated with lower risk of weight gain after 10 years of follow-up^26^. Third, insulin stimulates hydroxy methyl glutaryl (HMG) Co-A reductase, one of the rate-limiting enzymes in cholesterol syntheses by increasing rates of transcription^27^. Thus, skipping breakfast might induce higher LDL-C, which leads to atherosclerosis and eventually increases cardiometabolic risk^9^.

On the other hand, skipping breakfast can cause unhealthy eating habits at lunch or dinner. Individuals frequently skipping breakfast tended to eat a large amount at once during the rest of the day^28^. In a randomized controlled trial among obese people, those with morning fasting compared to having breakfast tended to have compensate calories throughout the rest of the day^29^. In addition, individuals who skipped breakfast rated higher appetite and hunger, less fullness, and increased ghrelin levels than those who consumed breakfast^30,31^. These changes in eating patterns can be related to overeating resulting in weight gain and insulin resistance.

In our study, breakfast skippers showed low overall diet quality; they were more likely to consume fast foods and foods containing high simple sugars but less likely to consume foods with high dietary fiber and micronutrients such as fruits and vegetables. Several prior studies reported that breakfast skippers consumed a significantly higher proportion of energy from fat^32^. A study using the US National Health and Nutrition Examination Survey data demonstrated that Healthy Eating Index was higher in young adults who consumed breakfast than breakfast skippers^33^. In our study, we adjusted for various established risk factors, including dietary intake of various healthy and unhealthy foods, as confounding factors. Association between breakfast skipping and metabolic syndrome was attenuated by adjusting for dietary intake of in model 3. Thus, we assumed that dietary quality acts as one of the meaningful causal factor in the relationship between the frequency of breakfast and metabolic syndrome. Moreover, skipping breakfast may partially be correlated with unhealthy behaviors including smoking, heavy alcohol consumption, and low physical activity, although we adjusted for these factors in our analyses.

We found that overall unhealthy meal patterns such as binge eating and having irregular meal were associated with metabolic syndrome, which was in line with prior studies. Brazilian Longitudinal Study of Adult Health study suggested that binge eating was associated with increased odds of metabolic syndrome (OR 1.66, 95% CI 1.44–1.75) as well as its components (hypertension and hypertriglyceridemia) through weight gain^34^. Having irregular meal was associated with insulin insensitivity by disturbing regular circadian variations of insulin secretion^35^.

Of the metabolic syndrome components, skipping breakfast was prominently associated with high BP. An experimental study in women aged 18–45 years (n = 65) found that breakfast skippers had higher circulating cortisol than breakfast eaters through stress-independent hyperactivity in hypothalamic–pituitary–adrenal axis^36^. Increased cortisol levels were found as a contributing factor to the development of hypertension (hazard ratio 1.23, 95% CI 1.04–1.44)^37^, which was stronger among younger individuals with a significant age interaction.

This study has some limitations. First, since the current study was cross-sectional, a causal relationship between breakfast frequency and metabolic syndrome could not be definitively established. Second, calorie intake was unknown due to a lack of information about the serving size of each food item. Third, our results are limited in generalizability to other ages or social groups because our study was performed among young university students. In addition, the prevalence of metabolic syndrome was low (2.6%) in our study. This can be attributed to the specific characteristics of our participants, who are highly educated young adults with a higher socio-economic status. Lastly, the information on meal patterns and dietary intake were obtained using self-reported questionnaire. Thus, there could exist inaccurate recall of their dietary behaviors.

In conclusion, higher frequency of skipping breakfast was positively associated with metabolic syndrome and its components (especially high BP). Our findings indicate that eating breakfast might be an important factor to reduce risk of metabolic syndrome in young adults. Further longitudinal studies are needed to confirm this association.

### Supplementary Information

Supplementary Tables.

## Data Availability

The datasets generated and/or analysed during the current study are available in the Korea National Health and Nutrition Examination Survey data and if requested, it can be anonymized and provided.
