# Supplementary material for: Association between breakfast frequency and metabolic syndrome among young adults in South Korea
Source: Sci Rep. 2023 Oct 6;13:16826. doi: 10.1038/s41598-023-43957-3 (PMC10558535; doi:10.1038/s41598-023-43957-3)
Supplement: Supplementary file 1 — Supplementary Tables. [file 41598_2023_43957_MOESM1_ESM.docx]

**Supplementary Materials**

**Supplementary Table 1.** Dietary intake among study participants according to breakfast consumption frequency

|  | **Total** | **Breakfast frequency, per week** | | | *P*-value |
| --- | --- | --- | --- | --- | --- |
|  |  | **7 days**  **(non-skipper)** | **4-6 days**  **(skipper for 1-3 days)** | **0-3 days**  **(skipper for 4-7 days)** |  |
|  | n = 12,302 | n = 2,152 (17.5%) | n = 3,169 (25.8%) | n = 6,981 (56.8%) |  |
| White rice, N (%) |  |  |  |  |  |
| <1/week | 1,219 (9.9) | 373 (17.3) | 313 (9.9) | 533 (7.6) | <0.001 |
| 1–4/week | 2,858 (23.2) | 426 (19.8) | 659 (20.8) | 1,773 (25.4) |  |
| ≥5/week | 8,225 (66.9) | 1,353 (62.9) | 2,197 (69.3) | 4,675 (67.0) |  |
| Brown rice/multigrain rice/beans, N (%) |  |  |  |  |  |
| <1/week | 4,058 (33.0) | 486 (22.6) | 849 (26.8) | 2,723 (39.0) | <0.001 |
| 1–4/week | 4,259 (34.6) | 555 (25.8) | 1,055 (33.3) | 2,649 (38.0) |  |
| ≥5/week | 3,985 (32.4) | 1,111 (51.6) | 1,265 (39.9) | 1,609 (23.1) |  |
| Bread/toast, N (%) |  |  |  |  |  |
| <1/week | 6,462 (52.5) | 934 (43.4) | 1,494 (47.1) | 4,034 (57.8) | <0.001 |
| 1–4/week | 4,664 (37.9) | 894 (41.5) | 1,281 (40.4) | 2,489 (35.7) |  |
| ≥5/week | 1,176 (9.6) | 324 (15.1) | 394 (12.4) | 458 (6.6) |  |
| Pizza, N (%) |  |  |  |  |  |
| <1/week | 11,082 (90.1) | 1,963 (91.2) | 2,887 (91.1) | 6,232 (89.3) | 0.012 |
| 1–4/week | 1,203 (9.8) | 185 (8.6) | 278 (8.8) | 740 (10.6) |  |
| ≥5/week | 17 (0.1) | 4 (0.2) | 4 (0.1) | 9 (0.1) |  |
| Hamburger/sandwich, N (%) |  |  |  |  |  |
| <1/week | 8,319 (67.6) | 1,576 (73.2) | 2,191 (69.1) | 4,552 (65.2) | <0.001 |
| 1–4/week | 3,755 (30.5) | 543 (25.2) | 906 (28.6) | 2,306 (33.0) |  |
| ≥5/week | 228 (1.9) | 33 (1.5) | 72 (2.3) | 123 (1.8) |  |
| Snack/cake/pie/sweets, N (%) |  |  |  |  |  |
| <1/week | 3,807 (31.0) | 702 (32.6) | 946 (29.9) | 2,159 (30.9) | 0.044 |
| 1–4/week | 6,409 (52.1) | 1,069 (49.7) | 1,711 (54.0) | 3,629 (52.0) |  |
| ≥5/week | 2,086 (17.0) | 381 (17.7) | 512 (16.2) | 1,193 (17.1) |  |
| Rice cake/Tteokbokki, N (%) |  |  |  |  |  |
| <1/week | 8,826 (71.7) | 1,608 (74.7) | 2,278 (71.9) | 4,940 (70.8) | 0.01 |
| 1–4/week | 3,374 (27.4) | 526 (24.4) | 864 (27.3) | 1,984 (28.4) |  |
| ≥5/week | 102 (0.8) | 18 (0.8) | 27 (0.9) | 57 (0.8) |  |
| Instant noodle/cup ramen, N (%) |  |  |  |  |  |
| <1/week | 6,339 (51.5) | 1,385 (64.4) | 1,717 (54.2) | 3,237 (46.4) | <0.001 |
| 1–4/week | 5,633 (45.8) | 734 (34.1) | 1,388 (43.8) | 3,511 (50.3) |  |
| ≥5/week | 330 (2.7) | 33 (1.5) | 64 (2.0) | 233 (3.3) |  |
| Fruits, N (%) |  |  |  |  |  |
| <1/week | 2,854 (23.2) | 187 (8.7) | 467 (14.7) | 2,200 (31.5) | <0.001 |
| 1–4/week | 5,850 (47.6) | 778 (36.2) | 1,513 (47.7) | 3,559 (51.0) |  |
| ≥5/week | 3,598 (29.3) | 1,187 (55.2) | 1,189 (37.5) | 1,222 (17.5) |  |
| Vegetables (except Kimchi), N (%) |  |  |  |  |  |
| <1/week | 1,303 (10.6) | 92 (4.3) | 217 (6.9) | 994 (14.2) | <0.001 |
| 1–4/week | 5,167 (42.0) | 667 (31.0) | 1,215 (38.3) | 3,285 (47.1) |  |
| ≥5/week | 5,832 (47.4) | 1,393 (64.7) | 1,737 (54.8) | 2,702 (38.7) |  |
| Milk and dairy products, N (%) |  |  |  |  |  |
| <1/week | 2,169 (17.6) | 215 (10.0) | 430 (13.8) | 1,524 (21.8) | <0.001 |
| 1–4/week | 5,915 (48.1) | 818 (38.0) | 1,416 (44.7) | 3,681 (52.7) |  |
| ≥5/week | 4,218 (34.3) | 1,119 (52.00) | 1,323 (41.8) | 1,776 (25.4) |  |
| Egg, N (%) |  |  |  |  |  |
| <1/week | 1,281 (10.4) | 112 (5.2) | 211 (6.7) | 958 (13.7) | <0.001 |
| 1–4/week | 6,824 (55.5) | 941 (43.7) | 1,648 (52.0) | 4,235 (60.7) |  |
| ≥5/week | 4,197 (34.1) | 1,099 (51.1) | 1,310 (41.3) | 1,788 (25.6) |  |
| Fatty fish, N (%) |  |  |  |  |  |
| <1/week | 7,165 (58.2) | 910 (42.3) | 1,657 (52.3) | 4,598 (65.9) | <0.001 |
| 1–4/week | 4,836 (39.3) | 1,132 (52.6) | 1,424 (44.9) | 2,280 (32.7) |  |
| ≥5/week | 301 (2.5) | 110 (5.1) | 88 (2.8) | 103 (1.5) |  |
| High-fat meat (pork belly/ribs/barbecue etc.), N (%) |  |  |  |  |  |
| <1/week | 3,531 (28.7) | 621 (28.9) | 875 (27.6) | 2,035 (29.2) | <0.001 |
| 1–4/week | 7,256 (59.0) | 1,199 (55.7) | 1,870 (59.0) | 4,187 (60.0) |  |
| ≥5/week | 1,515 (12.3) | 332 (15.4) | 424 (13.4) | 759 (10.9) |  |
| Processed meats (ham/sausage/bacon etc.), N (%) |  |  |  |  |  |
| <1/week | 4,922 (40.0) | 923 (42.9) | 1,246 (39.3) | 2,753 (39.4) | <0.001 |
| 1–4/week | 6,524 (53.0) | 1,060 (49.3) | 1,672 (52.8) | 3,792 (54.3) |  |
| ≥5/week | 856 (7.0) | 169 (7.9) | 251 (7.9) | 436 (6.3) |  |
| Sugared beverages, N (%) |  |  |  |  |  |
| <1/week | 4,360 (35.4) | 1,070 (49.7) | 1,209 (38.2) | 2,081 (29.8) | <0.001 |
| 1–4/week | 6,215 (50.5) | 895 (41.6) | 1,582 (49.9) | 3,738 (53.6) |  |
| ≥5/week | 1,727 (14.0) | 187 (8.7) | 378 (11.9) | 1,162 (16.7) |  |
| Coffee, N (%) |  |  |  |  |  |
| <1/week | 3,019 (24.5) | 599 (27.8) | 744 (23.5) | 1,676 (24.0) | <0.001 |
| 1–4/week | 2,934 (23.9) | 454 (21.1) | 756 (23.9) | 1,724 (24.7) |  |
| ≥5/week | 6,349 (51.6) | 1,099 (51.1) | 1,669 (52.7) | 3,581 (51.3) |  |

|  | **Breakfast frequency, per week** | | | *p* _trend_ |
| --- | --- | --- | --- | --- |
|  | **Non-skipper** | **Skipper for 1-3 days** | **Skipper for 4-7 days** |  |
| Metabolic syndrome defined by International Diabetes Federation criteria, | | | | |
| Model 1 | 1 (Ref) | 1.29(0.81-2.07) | 1.54(1.03-2.31) | 0.091 |
| Model 2 | 1 (Ref) | 1.27(0.78-2.04) | 1.48(0.98-2.24) | 0.162 |
| Model 3 | 1 (Ref) | 1.18(0.73-1.92) | 1.32(0.85-2.05) | 0.477 |
| Metabolic syndrome defined by World Health Organization criteria | | | | |
| Model 1 | 1 (Ref) | 0.94(0.70-1.28) | 1.37(1.06-1.77) | 0.001 |
| Model 2 | 1 (Ref) | 0.97(0.69-1.35) | 1.25(0.94-1.66) | 0.07 |
| Model 3 | 1 (Ref) | 0.95(0.68-1.33) | 1.17(0.86-1.58) | 0.25 |

**Supplementary Table 2**. Multivariable-adjusted odds ratios and 95% confidence intervals for association between breakfast consumption frequency and IDF-defined and WHO-defined metabolic syndrome

Model 1 adjusted for age, and sex

Model 2 adjusted for age, sex, alcohol, smoking, physical activity, and body mass index

Model 3 was adjusted for dietary intake of fruits, vegetables, milk and dairy products, high-fat meat, processed meat, and sugared beverages in addition to the covariates of model 2

**Supplementary Table 3**. Stratification analysis: association between breakfast consumption frequency and metabolic syndrome by sex

|  | **Breakfast frequency, per week** | | | *p* _trend_ | *P*_int_ |
| --- | --- | --- | --- | --- | --- |
|  | **Non-skipper** | **Skipper for 1-3 days** | **Skipper for 4-7 days** |  |  |
| Men |  | OR (95% CI) | |  | 0.926 |
| Model 1 | 1 (Ref) | 1.19(0.77-1.83) | 1.71(1.17-2.50) | 0.003 |  |
| Model 2 | 1 (Ref) | 1.30(0.82-2.06) | 1.65(1.10-2.47) | 0.034 |  |
| Model 3 | 1 (Ref) | 1.10(0.71-1.72) | 1.36(0.90-2.04) | 0.085 |  |
| Women |  | OR (95% CI) | |  |  |
| Model 1 | 1 (Ref) | 1.20(0.29-5.05) | 2.07(0.61-7.05) | 0.345 |  |
| Model 2 | 1 (Ref) | 1.11(0.25-4.83) | 1.53(0.43-5.43) | 0.712 |  |
| Model 3 | 1 (Ref) | 1.04(0.24-4.42) | 1.50(0.41-5.47) | 0.440 |  |

OR, odds ratio; CI, confidence interval

Model 1 adjusted for age, and sex

Model 2 adjusted for age, sex, alcohol, smoking, physical activity, and body mass index

Model 3 was adjusted for dietary intake of fruits, vegetables, milk and dairy products, high-fat meat, processed meat, and sugared beverages in addition to the covariates of model 2

**Supplementary Table 4**. Stratification analysis: association between breakfast consumption frequency and metabolic syndrome by physical activity

|  | **Breakfast frequency, per week** | | | *p* _trend_ | *P*_int_ |
| --- | --- | --- | --- | --- | --- |
|  | **Non-skipper** | **Skipper for 1-3 days** | **Skipper for 4-7 days** |  |  |
| Low physical activity | OR (95% CI) | | | | 0.217 |
| Model 1 | 1 (Ref) | 1.03(0.37-2.83) | 1.55(0.65-3.70) | 0.165 |  |
| Model 2 | 1 (Ref) | 1.56(0.52-4.64) | 1.74(0.68-4.42) | 0.264 |  |
| Model 3 | 1 (Ref) | 0.83(0.29-2.35) | 1.13(0.44-2.86) | 0.531 |  |
| Middle physical activity | OR (95% CI) | | | |  |
| Model 1 | 1 (Ref) | 1.52(0.89-2.60) | 2.11(1.31-3.39) | 0.001 |  |
| Model 2 | 1 (Ref) | 1.57(0.89-2.75) | 2.03(1.23-3.36) | 0.004 |  |
| Model 3 | 1 (Ref) | 1.45(0.84-2.49) | 1.76(1.06-2.91) | 0.025 |  |
| High physical activity | OR (95% CI) | | | |  |
| Model 1 | 1 (Ref) | 0.50(0.19-1.32) | 0.66(0.30-1.48) | 0.423 |  |
| Model 2 | 1 (Ref) | 0.52(0.19-1.46) | 0.69(0.29-1.65) | 0.498 |  |
| Model 3 | 1 (Ref) | 0.47(0.17-1.30) | 0.58(0.23-1.46) | 0.313 |  |

OR, odds ratio; CI, confidence interval

Model 1 adjusted for age, and sex

Model 2 adjusted for age, sex, alcohol, smoking, physical activity, and body mass index

Model 3 was adjusted for dietary intake of fruits, vegetables, milk and dairy products, high-fat meat, processed meat, and sugared beverages in addition to the covariates of model 2

**Supplementary Table 5.** Multivariable-adjusted odds ratios and 95% confidence intervals of metabolic syndrome according to frequency of binge eating

|  | **Binge eating, per week** | | |  |
| --- | --- | --- | --- | --- |
|  |  |  |  | *p* _trend_ |
|  | **<1 day** | **1-2 days** | **≥3 days** |  |
|  | n = 8,181 | n = 3,259 | n = 862 |  |
| N (%) | 145 (1.8) | 131 (4.0) | 43 (5.0) |  |
| Model 1 | 1 (Ref) | 2.26 (1.77-2.88) | 3.01 (2.11-4.29) | <0.001 |
| Model 2 | 1 (Ref) | 1.16 (0.90-1.51) | 1.24 (0.85-1.81) | 0.183 |
| Model 3 | 1 (Ref) | 1.14 (0.87-1.49) | 1.18 (0.80-1.74) | 0.292 |

Model 1 adjusted for age, and sex

Model 2 adjusted for age, sex, alcohol, smoking, physical activity, and BMI

Model 3 adjusted for age, sex, alcohol, smoking, physical activity, BMI, and dietary intake of fruits, vegetables, milk and dairy products, high-fat meat, processed meat, and sugared beverages

**Supplementary Table 6.** Multivariable-adjusted odds ratios and 95% confidence intervals of metabolic syndrome according to meal frequency

|  | **Meal frequency, per day** | | *p*-value |
| --- | --- | --- | --- |
|  | **Regular (3 meals)** | **Irregular (1 or 2 meals, others)** |  |
|  | n = 5,334 | n = 6,968 |  |
| N (%) | 114 (2.1) | 205 (2.9) |  |
| Model 1 | 1 (Ref) | 1.53 (1.21-1.93) | <0.001 |
| Model 2 | 1 (Ref) | 1.35 (1.05-1.74) | 0.019 |
| Model 3 | 1 (Ref) | 1.26 (0.97-1.64) | 0.084 |

Model 1 adjusted for age, and sex

Model 2 adjusted for age, sex, alcohol, smoking, physical activity, and BMI

Model 3 adjusted for age, sex, alcohol, smoking, physical activity, BMI, and dietary intake of fruits, vegetables, milk and dairy products, high-fat meat, processed meat, and sugared beverages
